# Genomic Epidemiology of *Salmonella* Isolated from Meat Products in China: Population Structure, Phylodynamics, and Antimicrobial Resistance

**DOI:** 10.3390/microorganisms14010191

**Published:** 2026-01-15

**Authors:** Shaoting Li, Wentao Ye, Yuheng Yang, Tianyue Zhu, Jiahao Ji, Miaomiao Chen, Yuxin Zheng, Hongmei Zhang, Qianwen Lu

**Affiliations:** 1 College of Biological and Pharmaceutical Science, Guangdong University of Technology, Guangzhou 510006, China; shaoting.li@gdut.edu.cn (S.L.);; 2 Central Maryland Research and Education Center, University of Maryland, Ellicott, MD 21042, USA

**Keywords:** foodborne pathogens, whole-genome sequencing, cgMLST, antimicrobial resistance genes, transmission dynamics

## Abstract

*Salmonella* is a major foodborne pathogen, and its increasing antimicrobial resistance poses a significant public health challenge. In this study, we conducted a comprehensive genomic epidemiological investigation of *Salmonella* isolates recovered from meat products across multiple provinces in China. A total of 141 isolates were collected and subjected to antimicrobial susceptibility testing and whole-genome sequencing. Core genome MLST and hierarchical clustering (HierCC) were performed using EnteroBase, while SNP phylogeny and phylodynamic analyses were conducted to characterize the evolutionary dynamics of *Salmonella* populations. The predominant serovars were Enteritidis and Infantis, with a high proportion of multidrug-resistant isolates. Potentially transferable plasmids carrying ARGs, such as *bla_CTX-M_*, *qnrS1*, *sul2*, and *mcr-1.1*, were frequently detected, indicating a risk of horizontal transfer during transmission. Genomic epidemiological investigation of our sequenced strains and their associated cgMLST HierCC clusters revealed both persistent *Salmonella* lineages, such as Enteritidis HC50-87 and Agona HC20-419, and emerging China-specific lineages, including Enteritidis HC20-10145 and Typhimurium HC50-2304. The estimated divergence times of these lineages mostly dated to the late mid-20th century, coinciding with the intensification of poultry farming in China. These findings highlight the power of genomic epidemiology in uncovering antimicrobial resistance patterns and transmission dynamics, underscoring the need for strengthened *Salmonella* surveillance.

## 1. Introduction

*Salmonella* has been identified as one of the leading causes of foodborne illnesses and is commonly found in animal-based foods, such as pork and poultry [1]. The advent of modern antibiotics has led to a gradual escalation in antibiotic resistance among *Salmonella*, particularly with the emergence of multidrug-resistant strains in meat, which has emerged as a substantial public health concern [2].

In recent years, a growing body of research from China has investigated the prevalence of *Salmonella* in retail markets. The majority of these studies have focused primarily on the antibiotic resistance and genotype diversity of *Salmonella* from livestock products [3]. The prevalence of *Salmonella* contamination has been observed to occur predominantly in retail markets. According to relevant domestic surveys, the contamination rate of *Salmonella* in retail markets in some regions of China has been investigated. For example, a study in Shanghai’s retail markets reported notably high *Salmonella* detection rates in pork (46.5%) and chicken (59.8%), while the rate in beef was substantially lower at 13.7% [4]. A high prevalence of *Salmonella* from chicken samples was reported in wholesale markets in Tianjin, China [5]. Furthermore, a study conducted in Jiangsu, China, reported the occurrence of *Salmonella* transmission from breeder chicken farms to commercial chicken farms, indicating the potential for vertical transmission within poultry populations [6]. The findings of these studies suggest that the contamination and transmission pathways of *Salmonella* in retail markets are intricate. Consequently, a comprehensive understanding of the genetic diversity, antibiotic resistance, and transmission pathways of *Salmonella* is essential for informing effective public health prevention and control strategies.

Whole-genome sequencing (WGS) has been demonstrated to facilitate the identification of various genetic elements associated with *Salmonella*, including its serovar, resistance genes, and virulence genes [7]. It also enables precise identification of genetic variations within the bacterial isolates, allowing more effective tracing of the origin and transmission routes of *Salmonella* [8]. The development and optimization of WGS have led to the widespread use of cgMLST (core genome multilocus sequence typing) and SNP (single-nucleotide polymorphism) methods in traceability studies of foodborne pathogens [9], providing improved insight into the population structure and epidemiological characteristics of bacterial isolates [10]. These methods also enable effective comparison of genomic data with public databases such as EnteroBase [11] and NCBI Pathogen Detection [12].

With the widespread application of WGS in foodborne pathogen investigation in China, an increasing number of studies have examined the prevalence, antimicrobial resistance, and genotype diversity of *Salmonella* isolates recovered from retail meats in various provinces, such as Zhejiang [13], Hebei [14], Sichuan [15], Shanxi [16], and Shandong [17]. These studies employed WGS with cgMLST or SNP analysis to ascertain the genetic heterogeneity of strains from disparate sources. The findings indicated a high degree of genetic variability among the *Salmonella* population in China. However, the phylogenetic relationships and the potential transmission pathways of *Salmonella* across different regions of China have been less frequently investigated in the context of global surveillance frameworks. Molecular source tracing based on global genomic big data facilitates accurate source tracking of *Salmonella*. Specifically, phylodynamic analysis, which integrates evolutionary and temporal information, has been increasingly employed to infer the transmission routes of *Salmonella* during its global spread [18,19] and to trace the dissemination of antimicrobial-resistant *Salmonella* populations driven by the acquisition of plasmid-mediated antimicrobial resistance [20,21].

The objective of this study is to conduct a systematic analysis of the isolation, genetic characteristics, and phylogenetic relationships of *Salmonella* from meat products across different regions of China and to investigate potential transmission sources and epidemic trends, thereby providing a theoretical basis to enhance pathogen surveillance and inform targeted prevention and control strategies.

## 2. Materials and Methods

### 2.1. Sample Collection

To ensure geographical representativeness across China, chicken samples were purchased from cold chain-transported, vacuum-sealed products originating from various provinces, including Guangdong (n = 54), Fujian (n = 26), Jiangsu (n = 16), Shandong (n = 16), Beijing (n = 11), Hebei (n = 10), Henan (n = 7), Liaoning (n = 3), and Anhui (n = 2), collected throughout all seasons between 2022 and 2023. Additionally, various fresh (non-packaged) meat and environmental samples were obtained from local wet markets in Guangzhou, Guangdong, including chicken (n = 19), pork (n = 12), surface swabs from meat cutting tables (n = 27), and wastewater (n = 21) from designated sampling sites from winter 2022 to spring 2023, for *Salmonella* detection and isolation. All samples were promptly transported to the laboratory and stored at 2–8 °C until analysis.

### 2.2. Isolation and Identification of Salmonella

*Salmonella* screening and isolation were performed according to the U.S. Food and Drug Administration (FDA) Bacteriological Analytical Manual (BAM) [22]. For pre-enrichment, each meat sample (25 g) was placed in 225 mL of Buffered Peptone Water (BPW, Hope Bio-technology Co., Ltd., Qingdao, China) and incubated at 37 °C overnight. Wastewater (10 mL) was mixed with 90 mL BPW, and surface swabs were placed in 100 mL BPW; these were also incubated at 37 °C overnight. Subsequently, 1 mL of the pre-enrichment culture was transferred into 10 mL of Rappaport-Vassiliadis (RV, Hope Bio-technology Co., Ltd., Qingdao, China) broth and incubated at 42 °C for 24 h. The enriched cultures were streaked onto Xylose Lysine Deoxycholate (XLD, Hope Bio-technology Co., Ltd., Qingdao, China) agar plates and incubated at 37 °C for 24 h for *Salmonella* detection. Positive colonies were purified by repeated streaking until pure isolates were obtained. The purified strains were preserved in Tryptic Soy Broth (TSB, Hope Bio-technology Co., Ltd., Qingdao, China) supplemented with 20% glycerol and stored at −80 °C until further use. In addition, *Salmonella* in meat samples was enumerated using the three-tube most probable number (MPN) method [23,24].

Genomic DNA was extracted from the purified *Salmonella* isolates using the Ezup Bacterial Genomic DNA Extraction Kit (Sangon Biotech, Shanghai, China). Preliminary identification of *Salmonella* was conducted by polymerase chain reaction (PCR) targeting the *invA* gene of *Salmonella* spp. [25].

### 2.3. Antimicrobial Susceptibility Testing

Antimicrobial susceptibility testing of the *Salmonella* isolates was evaluated using the broth dilution method. The resistance of the strains was assessed by determining the minimum inhibitory concentration (MIC). The MICs of 11 antimicrobial agents, including ampicillin (AMP), cefotaxime (CTX), ceftazidime (CAZ), cefoxitin (FOX), chloramphenicol (CHL), nalidixic acid (NAL), tetracycline (TET), colistin (CT), sulfamethoxazole (SUL), amikacin (AMI), and gentamicin (GEN) were determined, and the results were interpreted according to Clinical and Laboratory Standards Institute guidelines [26]. *Escherichia coli* ATCC 25922 was used as a quality control strain. Isolates showing resistance to at least three classes of antimicrobials were defined as multidrug resistance (MDR) [27].

### 2.4. Whole-Genome Sequencing and Bioinformatics Analysis

Whole-genome sequencing of the *Salmonella* isolates was performed in PE150 (paired ends mode, with 150 bp read length) using an Illumina NovaSeq 6000 (Illumina, San Diego, CA, USA). The sequencing reads were trimmed using Trimmomatic v0.39 [28] to remove adapter sequences and low-quality bases. Genomes were assembled with SPAdes v3.15 [29] and annotated with Prokka v1.14.6 [30]. The completeness and level of contamination of the assembled genomes were evaluated using CheckM v1.2.4 [31] with default parameters. Antimicrobial resistance genes (ARGs) were identified using ResFinder v4.0 [32] with an identity threshold of 90% and a minimum coverage of 80%. Plasmid typing was performed using the mob_typer function in MOB-suite v3.1.8 [33], with identity and coverage thresholds set at 85% and 60%, respectively. Pan-genome analysis was performed using Roary v3.13.0 [34]. A maximum likelihood phylogenetic tree was then constructed based on the core genome alignment using IQ-TREE v2.2.5 [35], and the resulting tree was visualized with TVBOT v2.6.0 [36]. Accession numbers for all genomes included in the analyses are provided in Table S1.

### 2.5. cgMLST and Hierarchical Clustering Analyses

A total of 141 *Salmonella* isolates were uploaded to the EnteroBase platform [37], where core genome multilocus sequence typing (cgMLST) and hierarchical clustering (HierCC) analyses [38] were performed using built-in algorithms. Based on cgMLST allelic profiles, HierCC grouped the isolates at various hierarchical levels according to allele differences, such as HC20 and HC50. Each HC level represents the maximum number of allele differences allowed within a cluster, enabling the formation of genetically related groups. To identify genetically related isolates of the *Salmonella* strains isolated in this study, the “Search Strains” function in EnteroBase was used to retrieve all genomes assigned to the same HC20 or HC50 clusters as of July 2024. In addition, the serovars and multilocus sequence types (MLSTs) of the isolates were obtained from the EnteroBase platform.

### 2.6. Selection of Representative Isolates from HierCC Clusters

Metadata of genomes belonging to the same HC20 or HC50 clusters were filtered to remove redundant isolates. Data processing was conducted using custom Python scripts. Isolates with incomplete metadata (e.g., missing country of origin, collection year, or BioSample accession) were excluded. Redundant isolates belonging to the same HC2 cluster were removed, since these were considered highly clonal. Within each HC2 cluster, only one isolate from the same country and collection year was retained. This strategy ensured the selection of representative and diverse isolates within each HC20 or HC50 cluster. Whole-genome sequencing data of the selected isolates were downloaded from the NCBI database using SRA Toolkit v3.0.7 based on their BioSample accessions (Table S2).

### 2.7. Phylogenetic Analysis of HierCC Clusters

Phylogenetic analysis was conducted using the selected representative isolates from each HC20 or HC50 cluster. Core single-nucleotide polymorphism (SNP) alignments were generated with Snippy v4.6.0 (https://github.com/tseemann/snippy (accessed on 1 May 2024)), using a genome from the same cluster as the reference. The resulting core SNP alignments were used to construct maximum likelihood (ML) phylogenetic trees using IQ-TREE v2.2.5 [35] with 1000 bootstrap replicates to assess branch support.

### 2.8. Temporal Signal Assessment and Phylodynamic Analysis

HC20 or HC50 associated with our isolates were chosen based on their suitability for phylodynamic analysis. The ML phylogenetic tree generated by IQ-TREE was imported into TempEst v1.5.3 [39], together with the collection years of the isolates, to assess the temporal signal of the HierCC clusters. Five isolates with residuals greater than 2 standard deviations from the regression line were considered temporal outliers and excluded. Temporal signal was assessed using root-to-tip regression analysis, applying a correlation coefficient (R^2^) threshold of 0.4 to indicate the presence of a temporal signal [19]. Phylodynamic analysis and ancestral sequence reconstruction were performed using TreeTime v0.11.1 [40]. The “coalescent skyline” option was applied to estimate changes in effective population size over time using a coalescent skyline model. Additionally, root-to-tip regression was performed to evaluate the molecular clock signal and estimate nucleotide substitution rates.

### 2.9. Statistical Analysis

Antibiotic resistance was encoded as 1 (resistant) and 0 (susceptible and intermediate), and the presence of ARGs was encoded as 1 (present) and 0 (absent). Correlations between binary phenotypic and genotypic variables were calculated using the Pearson correlation for binary data (Phi coefficient) with *p*-values computed via scipy.stats.pearsonr in Python v3.12.4. Associations with *p* < 0.05 were considered statistically significant. A Phi coefficient threshold of | Phi | > 0.4 was used to define strong correlations between binary phenotypic and genotypic variables [41].

## 3. Results

### 3.1. Isolation and Source Distribution of Salmonella Strains

**Figure 1:**
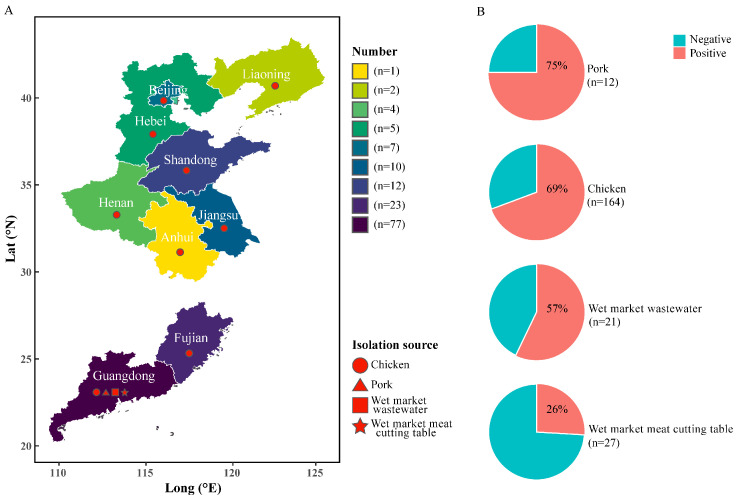
Source distribution and detection rates of *Salmonella* isolates. (**A**) Geographic source distribution of *Salmonella* isolates collected from nine provinces in China from 2022 to 2023. (**B**) Detection rates of *Salmonella* in different sample sources, including chicken, pork, wastewater, and meat cutting tables. n indicates the number of samples tested for each source.

From 2022 to 2023, this study isolated a total of 141 *Salmonella* strains (Table S1) across nine provinces in China, including 77 from Guangdong, 23 from Fujian, 12 from Shandong, 10 from Jiangsu, 7 from Beijing, 5 from Hebei, 4 from Henan, 2 from Liaoning, and 1 from Anhui (Figure 1). Of these strains, 113 were isolated from chicken samples, including 106 from packaged chicken collected across various provinces and 7 from fresh chicken meat collected at wet markets in Guangzhou. The remaining strains came from pork (9 strains), meat cutting tables (7 strains), and wastewater (12 strains) samples collected from local wet markets in Guangzhou, Guangdong. The overall detection rate was 75% in pork samples (9/12), 69% in chicken samples (113/164), 57% in wet market wastewater samples (12/21), and 26% in wet market meat cutting table samples (7/27). Of the 122 positive meat samples, 66 (54.1%) had a contamination level of 0–10 MPN/g, while 32 (26.2%) had a contamination of 10–100 MPN/g. Additionally, 24 (19.7%) samples had a contamination level exceeding 100 MPN/g (Table S3).

### 3.2. Distribution of Salmonella Serovars and MLST

The distribution of serovars and MLST of the *Salmonella* isolates is shown in Table 1. In total, 19 serovars were identified among the 141 *Salmonella* strains (Table S1). *Salmonella* Enteritidis and *Salmonella* Infantis were the two most predominant serovars, accounting for 37.6% (n = 53) and 14.9% (n = 21) of the total strains, respectively. Other detected serovars included London (n = 13), Kentucky (n = 11), Rissen (n = 11), Indiana (n = 6), Agona (n = 4), Typhimurium (n = 4), 1,4,[5],12:i:- (n = 3), Newport (n = 3), Thompson (n = 3), I B:e,h:- (n = 2), Corvallis (n = 1), Goldcoast (n = 1), Mbandaka (n = 1), Muenster (n = 1), Schwarzengrund (n = 1), Stanley (n = 1), and Weltevreden (n = 1).

Furthermore, *S.* Enteritidis and *S.* Infantis, both originating from chicken meat samples, were predominantly isolated from the Guangdong and Fujian provinces. In contrast, *S.* London and *S.* Rissen were isolated across all four sample types. A total of 21 MLST types were identified among the 141 *Salmonella* strains. Of these, *S.* Enteritidis was classified into two sequence types, ST11 and ST10481, while *S.* Kentucky was classified into ST198 and ST10418. The remaining serovars were each assigned to a single sequence type.

### 3.3. Antimicrobial Resistance and ARG Profiles in Salmonella

**Figure 2:**
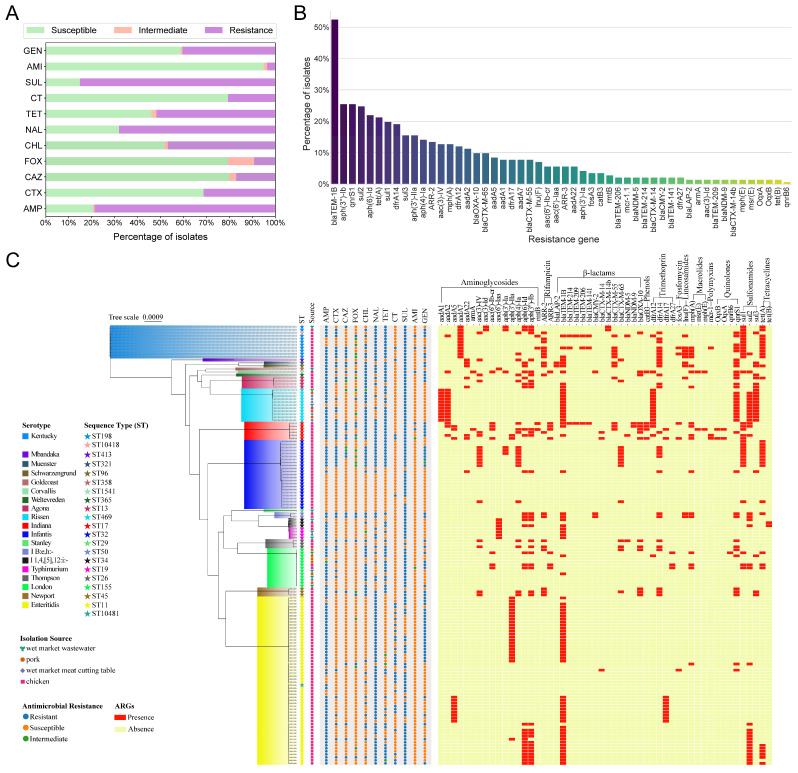
Antimicrobial resistance and resistance gene profiles in *Salmonella* isolates. (**A**) Percentage of *Salmonella* isolates exhibiting resistance to 11 Antibiotics. (**B**) Prevalence of antimicrobial resistance genes in *Salmonella* isolates. (**C**) Antimicrobial resistance and the presence of resistance genes across different *Salmonella* serovars. The phylogenetic tree was constructed based on core gene alignments generated by Roary. AMP: ampicillin, CTX: cefotaxime, CAZ: ceftazidime, FOX: cefoxitin, CHL: chloramphenicol, NAL: nalidixic acid, TET: tetracycline, CT: colistin, SUL: sulfamethoxazole, AMI: amikacin, and GEN: gentamicin.

The results of antimicrobial susceptibility testing (Table S4) revealed that the *Salmonella* isolates exhibited the highest resistance to sulfamethoxazole (SUL) and ampicillin (AMP), with resistant strains accounting for approximately 85.1% and 78.7% of the total, respectively (Figure 2A). In contrast, the isolates demonstrated the greatest susceptibility to amikacin, with about 95.0% of strains remaining sensitive. Additionally, the isolates showed relatively high sensitivity to cephalosporin antibiotics, with approximately 68.8%, 80.1%, and 79.4% of strains being sensitive to cefotaxime (CTX), ceftazidime (CAZ), and cefoxitin (FOX), respectively (Figure 2A).

Additionally, this study analyzed the antimicrobial resistance gene profiles of all isolates based on whole-genome sequencing data (Figure 2B and Table S5). A total of 52 different resistance genes were identified. Among β-lactamase genes, *bla_TEM-1B_* was the most prevalent, accounting for 52.5%. For aminoglycoside resistance genes, *aph(3′)-Ib* accounted for the highest percentage at 25.5%. Two rifamycin-like resistance genes, *ARR-2* and *ARR-3*, were present at percentages of approximately 13.5% and 5.7%, respectively. Among the four methicillin-like resistance genes, *dfrA14* was the most common, comprising 19.1%. The macrolide resistance gene *mph(A)* had a prevalence of 12.8%. For quinolones, sulfonamides, and tetracyclines, the most frequently detected genes were *qnrS1* (25.5%), *sul2* (24.8%), and *tet(A)* (21.3%), respectively. Additionally, one resistance gene was identified for each of the following antimicrobial classes: *caB3* (phenicols), *fosA3* (fosfomycins), *lun(F)* (lincosamides), and *mcr-1.1* (polymyxins).

Analysis of antimicrobial resistance and the presence of resistance genes across various *Salmonella* serovars revealed that most isolates exhibited multidrug resistance (Figure 2C). Interestingly, six *S.* Indiana isolates exhibited consistent resistance to six antibiotics: ampicillin, cefotaxime, chloramphenicol, nalidixic acid, tetracycline, and sulfamethoxazole. These *S.* Indiana isolates also carried a wide variety of resistance genes. Eleven isolates of *S.* Kentucky were resistant to nalidixic acid, tetracycline, and sulfamethoxazole, with all strains harboring the *aadA7* and *sul1* resistance genes. *S.* Enteritidis strains displayed higher resistance rates to ampicillin and nalidixic acid but exhibited low resistance gene diversity, predominantly carrying *aph(3′)-IIa* and *bla_TEM-1B_*. More than half of the *S.* Infantis strains were susceptible to ten antibiotics, with the exception of sulfamethoxazole, and some did not carry any resistance genes. Furthermore, all *S.* Rissen isolates, collected from multiple sources, consistently carried *aadA1*, *aadA2*, *bla_TEM-1B_*, *dfrA12*, and *sul3* resistance genes.

Correlation analysis between phenotypic resistance and ARGs revealed several strong and statistically significant associations (|Phi| > 0.4, *p* < 0.05) (Table S6). The strongest was *rmtB* with amikacin resistance (Phi = 0.89, *p* < 0.001). Several *bla_TEM_* variants, *mph(E)*, *msr(E)*, and *armA* also correlated with amikacin resistance (Phi = 0.63–0.77, *p* < 0.001). *bla_CTX-M-55_* showed strong associations with ceftazidime and cefotaxime resistance (Phi = 0.43–0.57, *p* < 0.001), while *bla_NDM-5_* and *bla_CMY-2_* correlated with cefoxitin resistance (Phi = 0.46, *p* < 0.001). Additional significant correlations (Phi > 0.4, *p* < 0.001) were found between *tet(A)* and tetracycline, *sul1* with tetracycline and cefotaxime, *aph(3′)-IIa* with gentamicin, and *bla_TEM-1B_* with ampicillin.

### 3.4. Identification of Plasmids in the Salmonella Isolates

**Figure 3:**
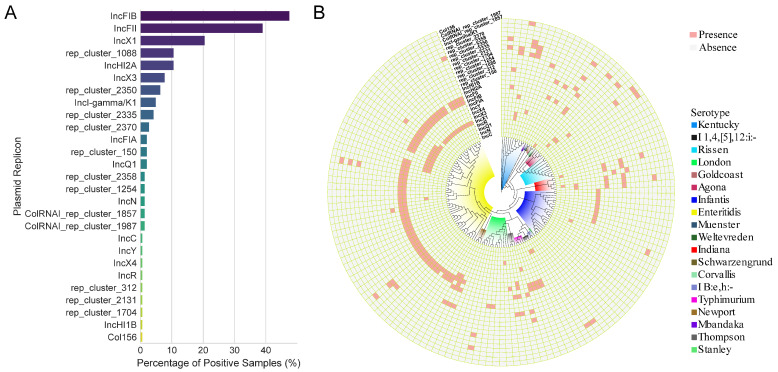
The identification of plasmid replicon types in *Salmonella* isolates. (**A**) Percentage of plasmid replicon types identified in *Salmonella* isolates. (**B**) Presence of plasmid replicon types across different *Salmonella* serovars.

Whole-genome sequences of 141 *Salmonella* strains were analyzed to identify plasmids and assess their carriage within the isolates (Figure 3A and Table S7). The assembled genomes were evaluated using CheckM to confirm completeness and the absence of contamination (Table S8). A total of 27 plasmids with identified replicon types were detected. The three most frequent replicon types were IncFIB, IncFII, and IncX1, accounting for 47.5%, 39.0%, and 20.6% of the strains, respectively. The presence of plasmids of different *Salmonella* serovars was analyzed (Figure 3B). The results showed that the three replicon types, IncFIB, IncFII, and IncX1, were predominantly present in *S.* Enteritidis strains. In addition, the IncFIB plasmid was detected in nine of the 23 *S.* Infantis strains. The IncFIB and IncFII plasmids were identified in all four *S.* Typhimurium strains. Interestingly, no plasmid replicon types were detected in the *S.* Rissen strains.

### 3.5. Characterization of ARG-Containing Plasmids and Mobility Prediction

To investigate whether antimicrobial resistance genes in different *Salmonella* serovars were plasmid-borne and to predict their mobility, plasmid prediction was performed using MOB-suite, which characterized the associated relaxase types, mating pair formation (MPF) systems, and origins of transfer (oriT) potentially involved in plasmid-mediated transfer. As shown in Table 2, plasmid-associated ARGs were identified within the predicted plasmid replicon sequences of eight *Salmonella* serovars.

Among these plasmids, only one relaxase type (MOBP) and three conjugative systems (MPF_F, MPF_T, and MPF_I) were present. Two types of origins of transfer, MOBP and MOBF, were also detected. In *S.* Enteritidis, ARGs were distributed across four plasmid replicon types (IncX1, IncX3, IncFIB, and IncFII), most of which were classified as conjugative or mobilizable plasmids. Notably, the *mcr-1.1* gene was identified on a conjugative IncX4 plasmid in one *S.* Kentucky isolate. Additionally, the *qnrS1* gene was detected on mobilizable plasmids in one *S.* Agona isolate and one *S.* Corvallis isolate. For *S.* London, *S.* Indiana, and *S.* 1,4,[5],12:i:-, although ARGs were identified within replicon-predicted plasmid sequences, no relaxase, MPF system, nor oriT were detected.

### 3.6. Estimation of Divergence Times for cgMLST HierCC Clusters of Salmonella Isolates

To investigate the evolutionary relationships and transmission routes of *Salmonella* isolates from different regions of China, 11 HC20 and 15 HC50 clusters associated with our strains were selected based on their suitability for phylodynamic analysis to infer divergence times of related isolates (Table 3). As shown in Table 3, a majority of the analyzed cgMLST HierCC clusters (19/26) exhibited an R^2^ ≥ 0.4, indicating a strong temporal signal. Seven clusters fell below the 0.4 threshold (e.g., *S.* Enteritidis HC20-374, *S.* Infantis HC50-36, and *S.* London HC50-37), suggesting weak or absent temporal structure for those lineages. These results demonstrated that most selected clusters possessed a sufficient temporal signal to support reliable estimation of divergence time.

Among all clusters, *S.* Typhimurium HC50-2304 demonstrated a strong temporal signal, with an R^2^ as high as 0.90. *S.* Weltevreden HC50-314 exhibited the earliest estimated divergence time, with an inferred MRCA in 1897 and a corresponding R^2^ value of 0.63. The latest inferred MRCA across all clusters was observed in *S.* Infantis HC20-343, dated to 2008, with an R^2^ of 0.73. In the 16 cgMLST HierCC clusters with strong temporal signals (R^2^ ≥ 0.4), five clusters—Enteritidis HC50-87 and HC20-87, Agona HC20-419 and HC50-29, and Muenster (HC50-47)—were estimated to have diverged in the mid-20th century (1940–1969), reflecting long-standing and persistent *Salmonella* lineages. The largest group, comprising eight clusters of diverse serovars, including Kentucky, Rissen, Indiana, Typhimurium, 1,4,[5],12:i:-, Newport, Thompson, and Corvallis, was estimated to have emerged during the late 20th century (1970–1999), suggesting extensive diversification of *Salmonella* lineages during this period. Finally, three clusters (Enteritidis HC20-7228, HC20-10145, and Infantis HC20-343) showed more recent divergence in the early 21st century (2000 onward), suggesting ongoing expansion and recent emergence of these lineages.

### 3.7. Phylodynamic Analysis for cgMLST HierCC Clusters of Salmonella Isolates

**Figure 4:**
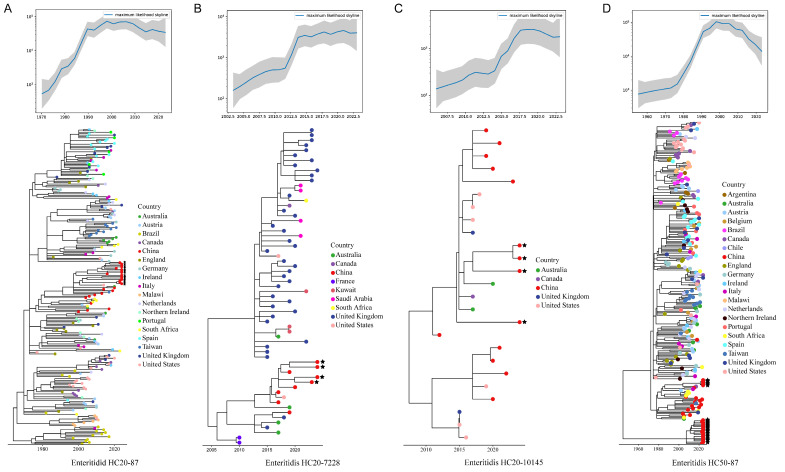
Ancestral sequence reconstruction and effective population size dynamics of (**A**) *S.* Entertidis HC20-87, (**B**) *S.* Entertidis HC20-7228, (**C**) *S.* Entertidis HC20-10145, and (**D**) *S.* Entertidis HC50-87. Red circles with star symbols indicate isolates from this study.

Phylodynamic analysis was conducted on the selected HC20/HC50 clusters to investigate the evolutionary relationships and transmission dynamics of *Salmonella* isolates examined in this study. As shown in Figure 4A, 14 sequenced *S.* Enteritidis isolates clustered within HC20-87, which diverged around 1963 (R^2^ = 0.68; Table 3). The effective population size of this lineage increased from 1970 to 2000 and stabilized thereafter. Interestingly, all Chinese strains fell within a single clade, comprising sequenced isolates from various provinces, including Guangdong, Shandong, Hebei, Jiangsu, Beijing, and Fujian. These were phylogenetically related to several strains from South Africa, Malawi, and Australia. Four sequenced *S.* Enteritidis isolates (from Guangdong and Liaoning) clustered within the HC20-7228 cluster (Figure 4B), which diverged into two major clades predominantly composed of isolates from China and the UK, with an MRCA estimated around 2005 (R^2^ = 0.74; Table 3). Its effective population size increased from 2002 and stabilized after 2015. The phylogeny of this cluster further revealed that the sequenced strains shared recent common ancestry with a US isolate from 2017. Another associated *S.* Enteritidis cluster, HC20-10145 (Figure 4C), was estimated to have diverged around 2004 (R^2^ = 0.78; Table 3), with an increasing trend in effective population size until 2017. This cluster comprised relatively few strains, most of which were from China. The sequenced strains (from Henan, Beijing, and Guangdong) shared recent common ancestry with strains from other regions of China, Australia, the UK, and the US. Notably, all the aforementioned *S.* Enteritidis clusters were nested within the broader HC50-87 cluster (Figure 4D), which diverged around 1952 (R^2^ = 0.67; Table 3). Additionally, the HC50-87 cluster also included HC20-5389, which comprised 19 additional sequenced strains from this study (mostly from Fujian, Guangdong, and Beijing) and other isolates predominantly of Chinese origin.

**Figure 5:**
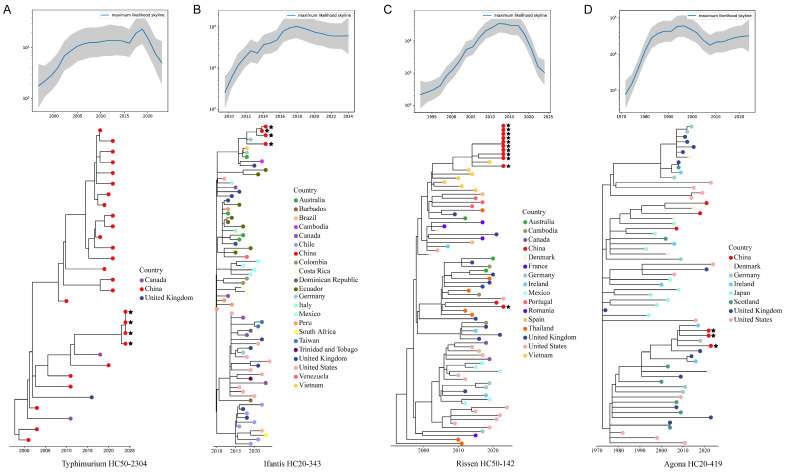
Ancestral sequence reconstruction and effective population size dynamics of (**A**) *S.* Typhimurium HC50-2304, (**B**) *S.* Infantis HC20-343, (**C**) *S.* Rissen HC50-142, and (**D**) *S.* Agona HC20-419. Red circles with star symbols indicate isolates from this study.

Four sequenced *S.* Typhimurium isolates (all from Guangdong) clustered within HC50-2304, which exhibited the strongest temporal signal among all clusters (R^2^ = 0.90; Table 3). Despite clustering at the HC50 level, this group contains few isolates in the database, nearly all originating from China. As shown in Figure 5A, these strains shared recent common ancestry with three isolates from Canada and the UK. HC50-2304 likely represents a recently emerged, China-specific lineage, post-2000, and warrants further investigation.

Among all HierCC clusters, *S.* Infantis HC20-343 exhibited the most recent divergence time, estimated around 2008 (R^2^ = 0.73; Table 3). Four strains from this study (from Guangdong and Jiangsu) clustered within the same evolutionary branch, closely related to a German isolate from 2019 (Figure 5B). Notably, these four were the only Chinese strains presented in the HC20-343 phylogeny.

All 11 sequenced *S.* Rissen isolates (all from Guangdong) belonged to the HC50-142 cluster, which diverged around 1983 (R^2^ = 0.68; Table 3). As shown in Figure 5C, the effective population size of this cluster has declined since 2015. Ten of the sequenced strains clustered within the same evolutionary branch and shared recent common ancestry with isolates from Vietnam, while the remaining strain was more closely related to a 2011 isolate from Thailand.

Three sequenced *S.* Agona isolates from this study (all from Guangdong) clustered within HC20-419, which diverged around 1967 (R^2^ = 0.77; Table 3). As shown in Figure 5D, this cluster experienced rapid population expansion in the late 20th century, stabilizing around 2005. The sequenced strains were closely related to isolates from Ireland (2010) and Germany (2015), forming a clade primarily composed of isolates from European countries. The HC20-419 strains, together with another *S.* Agona strain belonging to HC20-375395, were nested within HC50-29, with an estimated divergence time of 1942 (R^2^ = 0.64; Table 3).

Other associated HierCC clusters with strong temporal signals (R^2^ > 0.6) included *S.* Schwarzengrund HC50-54 (MRCA = 1936, R^2^ = 0.72) and *S.* Weltevreden HC50-314 (MRCA = 1897, R^2^ = 0.54). Each of these clusters contained only one isolate from this study and included isolates from multiple countries (Table S2), suggesting broader geographic distribution. Both clusters exhibited older divergence times, indicating they may represent long-established global lineages.

## 4. Discussion

Our study highlights the prevalence, antimicrobial resistance, and dissemination of circulating *Salmonella* serovars in meat products in China. To build on these findings, systematic, multi-year surveillance across diverse food matrices and regions is needed to better capture geographic variation and transmission pathways. Monitoring emerging lineages of *Salmonella* is essential due to their potential multidrug resistance and public health impact. Strengthening integrated One Health approaches that link human, animal, livestock products, and environmental surveillance is crucial to further curb the spread of *Salmonella* and antimicrobial resistance and to better inform food safety policies.

The dissemination and prevalence of major *Salmonella* serovars remain a significant public health and food safety concern globally. In China, *S.* Enteritidis and *S.* Typhimurium have historically been the most prevalent serovars. Wang et al. [42] reported the isolation of over 30,000 *Salmonella* strains across China from 2006 to 2019, including more than 20,000 from human clinical samples, with *S.* Enteritidis and *S.* Typhimurium identified as the predominant serovars. In the present study, chicken samples collected from multiple regions in China from 2022 to 2023 revealed *S.* Enteritidis and *S.* Infantis as the dominant serovars. This finding was consistent with the report by Zhao et al. [43], who investigated the prevalence of *Salmonella* in broiler farms, slaughterhouses, and markets in Shandong Province of China in 2022. These results indicated that, while *S.* Enteritidis remains a predominant serovar, *S.* Infantis is increasingly prevalent, particularly among poultry-derived isolates in China.

The *Salmonella* isolated in this study exhibited high resistance rates to sulfamethoxazole (~85%) and ampicillin (~79%) (Figure 2A), consistent with the findings of Zhao et al. [43]. Notably, discrepancies were observed between the results of antimicrobial susceptibility testing and the presence of corresponding antimicrobial resistance genes. For instance, while most isolates were phenotypically resistant to sulfamethoxazole, not all harbored known sulfonamide resistance genes (e.g., *sul1*, *sul2*, or *sul3*). This suggests that antimicrobial resistance is influenced by factors beyond the presence of currently recognized resistance genes. Schrader et al. [44] described this discrepancy as phenotypic resistance, which is often associated with bacterial states such as tolerance, persistence, or heteroresistance. Balaban et al. [45] suggested that this phenomenon may be related to the metabolic state of the bacterial cells. These findings highlight the importance of considering both genotypic and phenotypic mechanisms when investigating antimicrobial resistance.

Three plasmid replicon types, IncFIB, IncFII, and IncX1, were the most frequently detected, predominantly among *S.* Enteritidis isolates. These plasmids carried multiple clinically relevant ARGs such as *bla_TEM-1B_*, *sul2*, *tet(A)*, and *qnrS1*. Notably, mobility prediction indicated that many of these plasmids were either conjugative or mobilizable (Table 2), suggesting a significant risk for the horizontal dissemination of antimicrobial resistance. These findings are consistent with previous reports [46,47,48] about the prevalence and resistance profiles of *Salmonella* isolates from meat products across various regions of China, in which IncFIB, IncFII, and IncX1 plasmids were frequently identified as key carriers of antimicrobial resistance determinants.

Plasmids can be classified as conjugative, mobilizable, or non-mobilizable based on the presence of key mobility determinants—relaxase, mating pair formation (MPF) systems, and origin of transfer (oriT)—which are critical for mediating plasmid transfer [33]. Most conjugative plasmids in our dataset encoded all essential components required for autonomous plasmid transfer between bacterial cells. For example, the IncX1, IncX3, IncFIB, and IncFII plasmids identified in *S.* Enteritidis were predominantly classified as conjugative (Table 2). In contrast, strains identified with mobilizable plasmids, such as *S.* Corvallis and *S.* Agona, lacked MPF components and thus require a co-resident conjugative plasmid to mediate their transfer via the MPF-encoded channel [49]. In our study, plasmids in *S.* Indiana, *S.* 1,4,[5],12:i:-, and *S.* London were classified as non-mobilizable, suggesting a lack of essential components for conjugative transfer. Notably, colistin resistance genes of the *mcr* family were identified in colistin-resistant *S.* Kentucky and *S.* Indiana isolates (Figure 2C). In *S.* Kentucky, the *mcr-1.1* gene was located on a conjugative IncX4 plasmid (Table 2). The presence of *mcr-1*-bearing IncX4 plasmids has also been reported in other *Salmonella* serovars, including *S.* Typhimurium [50] and *S.* Infantis [51], raising serious concern due to their role in promoting the spread of resistance to colistin and other antimicrobials. Among two *S.* Indiana isolates harboring *mcr-1.1*, the gene was located on predicted plasmid sequences; however, the replicon types of these plasmids could not be determined. Notably, *mcr-1*-carrying plasmids have also been identified in *S.* Indiana isolates from retail chicken meats [52] and poultry slaughterhouses [53] in China. These findings suggest that conjugative or mobilizable plasmids harboring clinically relevant ARGs in *Salmonella* isolates are circulating across various regions of China. This underscores the need for further investigation and enhanced surveillance of such plasmids to better understand the horizontal transmission of resistance genes.

In our study, the sequenced strains were initially analyzed using cgMLST typing and HierCC clustering to identify closely related isolates from the database. A core SNP-based phylogenetic tree was then constructed to infer transmission routes, while divergence times and the most recent common ancestor of each HierCC cluster were estimated through phylodynamic analysis. Interestingly, the majority of identified clusters exhibited divergence in the late 20th century (Table 3), likely coinciding with the rise of industrialized poultry farming in China. Although China has a long agricultural history, farming was predominantly small-scale and backyard-based until the late 1970s [54]. The onset of industrialization prompted a rapid transition to intensive, large-scale poultry production, a development that has been linked to the global dissemination of *Salmonella* [19].

Specifically, the most prevalent *Salmonella* population identified in this study, *S.* Enteritidis HC50-87 (Figure 4D), represents a historically persistent population (MRCA = 1952) that has spread globally. Our results further revealed several emerging localized *S.* Enteritidis populations in China, including HC20-7228 and HC20-10145 (Figure 4B,C), with divergence times after 2000. *S.* Enteritidis HC50-87 is a subpopulation of ST11, which is currently the most common genotype associated with *S.* Enteritidis isolates. This lineage has been reported to carry IncX1, IncFIB, and IncFII plasmids linked to MDR, including clinically relevant ARGs such as *bla_CTX-M_* and *dfrA17* [55], both of which were also detected in this study. *S.* Agona HC20-419 (Figure 5D) also appears to be a persistent population (MRCA = 1967), predominantly composed of isolates from European countries, particularly the United Kingdom [56]. The *S.* Agona HC20-419 strains identified in this study warrant special attention, as they were found to be multidrug-resistant, carrying plasmids harboring multiple ARGs, including *bla_CTX-M_*, *qnrS1*, *sul3*, and *tet(A)*—potentially acquired through horizontal gene transfer during transmission [57].

*S.* Typhimurium HC50-2304 (Figure 5A) is a newly expanding population that diverged around 2000 and appears to be specifically associated with isolates from China. In addition to the Guangdong isolates collected in this study, it also included strains from multiple provinces, such as Shanghai, Zhejiang, and Shanxi. *S.* Infantis HC20-343 (Figure 5B) is another newly emerged lineage (MRCA = 2008) that warrants attention. Although HC20-343 isolates were widespread globally, only four strains from China were identified—all of which were collected in this study from Guangdong and Jiangsu. Notably, the four isolates all carried a pESI-like plasmid (Tables S5 and S7), which has raised increasing global concern due to its carriage of multiple clinically relevant ARGs, including *bla_CTX-M_*, *aph(3′)-Ia*, *dfrA14*, *floR*, *sul1*, and *tet(A)* [58]. These emerging lineages warrant further attention due to their geographic spread and potential public health relevance.

Apart from chicken meat, *S.* Rissen HC50-142 (Figure 5C) was frequently detected in pork products and meat cutting tables from wet markets in Guangzhou, China. *S.* Rissen is one of the important *Salmonella* serovars linked with swine products in numerous countries and is commonly associated with food contamination along the swine production chain. A recent study has reported the widespread dissemination of antimicrobial resistance in *S.* Rissen in China [59]. Strains classified as *S.* Weltevreden HC50-314 shared a recent common ancestor with Philippine strains (Table S2). This aligns with findings by Li et al. [60], who reported strong phylogenetic relatedness between *S.* Weltevreden strains from southern coastal China and Southeast Asia. In recent years, *S.* Weltevreden has attracted increasing attention in South and Southeast Asia, particularly due to its association with zoonotic infections via contaminated seafood [61,62].

The observed temporal patterns highlight the robustness of our phylodynamic framework in capturing both the historical persistence and recent emergence of *Salmonella* populations. However, strains classified as *S.* Infantis HC50-36, *S.* London HC50-37, and *S.* Goldcoast HC20-13193 exhibited weak temporal signals, likely due to the under-representativeness of collected isolates over their extended evolutionary histories. These findings underscore the need for sustained and coordinated efforts to enhance surveillance strategies for *Salmonella* and other pathogenic bacteria.

Several limitations of this study should be acknowledged. First, the origin of the samples was heterogeneous in both location and source, which may partially bias the observed prevalence of *Salmonella* serovars across China. Second, the two-year sampling period may not fully capture seasonal or annual variations in *Salmonella* prevalence, limiting the ability to generalize the findings over longer time scales. Finally, the reliance on short-read sequencing may limit the resolution of certain plasmids or mobile genetic elements carrying antimicrobial resistance genes, as these regions may not be fully sequenced.

## 5. Conclusions

This study provides a comprehensive overview of *Salmonella* prevalence, antimicrobial resistance, plasmid carriage, and evolutionary dynamics across multiple provinces in China. *S.* Enteritidis and *S.* Infantis were the dominant serovars among the collected isolates, with high rates of resistance to sulfamethoxazole and ampicillin. Conjugative and mobilizable plasmids carrying clinically relevant ARGs, including *bla_CTX-M_*, *qnrS1*, *sul2*, and *mcr-1.1*, were frequently detected, highlighting the risk of horizontal gene transfer. Phylodynamic analysis revealed both historically persistent and recently emerging *Salmonella* lineages, emphasizing the need for continued surveillance. These findings underscore the importance of integrated monitoring and targeted interventions to control the dissemination of *Salmonella* and multidrug resistance.

## Figures and Tables

**Table 1:** Distribution of *Salmonella* serovars and MLST.

Serovar	ST (n)	Source Type (n)	Region (n)
Enteritidis	11 (52) 10481 (1)	Poultry (53)	Fujian (15), Guangdong (11), Jiangsu (8), Beijing (6), Shandong (5), Hebei (3), Henan (3), Liaoning (2)
Infantis	32 (21)	Poultry (21)	Guangdong (12), Fujian (5), Anhui (1), Hebei (1), Jiangsu (1), Shandong (1)
London	155 (13)	Swine (6), wet market wastewater (4), poultry (2), wet market food contact surface (1)	Guangdong (13)
Kentucky	198 (10) 10418 (1)	Poultry (10), wet market wastewater (1)	Guangdong (7), Shandong (4)
Rissen	469 (11)	Wet market food contact surface (5), swine (3), wet market wastewater (2), poultry (1)	Guangdong (11)
Indiana	17 (6)	Poultry (6)	Guangdong (4), Hebei (1), Shandong (1)
Agona	13 (4)	Poultry (4)	Guangdong (3), Shandong (1)
Typhimurium	19 (4)	Poultry (3), wet market food contact surface (1)	Guangdong (4)
I 1,4,[5],12:i:-	34 (3)	Poultry (2), wet market wastewater (1)	Guangdong (3)
Newport	45 (3)	Poultry (3)	Fujian (2), Guangdong (1)
Thompson	26 (3)	Poultry (3)	Guangdong (2), Jiangsu (1)
I B:e,h:-	50 (2)	Poultry (2)	Guangdong (2)
Corvallis	1541 (1)	Poultry (1)	Beijing (1)
Goldcoast	358 (1)	Wet market wastewater (1)	Guangdong (1)
Mbandaka	413 (1)	Poultry (1)	Fujian (1)
Muenster	321 (1)	Poultry (1)	Guangdong (1)
Schwarzengrund	96 (1)	Poultry (1)	Henan (1)
Stanley	29 (1)	Wet market wastewater (1)	Guangdong (1)
Weltevreden	465 (1)	Poultry (1)	Guangdong (1)

**Table 2:** Plasmid replicon types, associated ARGs, and predicted mobility in *Salmonella* isolates.

Serovar	Replicon Type (n)	Relaxase Type (n)	MPF Type (n)	oriT Type (n)	Predicted Mobility	Resistance Gene (n)
Enteritidis	IncX1 (22)	MOBP (6)	MPF_F (6)	MOBP (6)	conjugative	*aph(6)-Id* (6) *aph(3″)-Ib* (6) *bla_TEM-1B_* (6) *sul2* (6)
		MOBP (6)	—	MOBP (6)	mobilizable	*aph(6)-Id* (6) *aph(3″)-Ib* (6) *bla_TEM-1B_* (1) *sul2* (6) *tet(A)* (6)
		MOBP (10)	MPF_T (10)	MOBP (9)	conjugative	*aadA5* (9) *bla_TEM-1B_* (9) *dfrA17* (9) *qnrS1* (1)
	IncX3 (9)	MOBP (9)	MPF_T (9)	MOBP (9)	conjugative	*aadA5* (9) *bla_TEM-1B_* (9) *dfrA17* (9)
	IncFIB (8)	MOBP (6)	MPF_F (6)	MOBP (6)	conjugative	*aph(6)-Id* (6) *aph(3″)-Ib* (6) *bla_TEM-1B_* (6) *sul2* (6)
		—	MPF_F (2)	—	non-mobilizable	*bla_TEM-1B_* (2)
	IncFII (8)	MOBP (6)	MPF_F (6)	MOBP (6)	conjugative	*aph(6)-Id* (6) *aph(3″)-Ib* (6) *bla_TEM-1B_* (6) *sul2* (6)
		—	MPF_F (2)	—	non-mobilizable	*bla_TEM-1B_* (2)
London	IncI-gamma/K1 (1)	—	—	—	non-mobilizable	*bla_TEM-1B_* (1)
Kentucky	IncX4 (1)	MOBP (1)	MPF_T (1)	—	conjugative	*mcr-1.1* (1)
Agona	IncX1 (1)	—	—	MOBF (1)	mobilizable	*aph(6)-Id* (1) *aph(3″)-Ib* (1) *qnrS1* (1)
1,4,[5],12:i:-	IncQ1 (2)	—	—	—	non-mobilizable	*aph(6)-Id* (2) *aph(3″)-Ib* (2) *sul2* (2)
I b:e,h:-	IncI-gamma/K1 (2)	MOBP (2)	MPF_I (2)	MOBP (2)	conjugative	*bla_CMY-2_* (2)
Indiana	IncX1 (1)	—	—	—	non-mobilizable	*aph(3′)-IIa* (1)
	rep_cluster_1088 (1)	—	—	—	non-mobilizable	*mcr-1.1* (1)
	rep_cluster_1254 (1)	—	—	—	non-mobilizable	*armA* (1) *aac(6′)-Ib-cr* (1) *bla_OXA-1_* (1) *msr(E)* (1) *mph(E)* (1) *catB3* (1) *ARR-3* (1) *sul1* (1)
Corvallis	rep_cluster_1254 (1)	—	—	—	non-mobilizable	*armA* (1) *msr(E)* (1) *mph(E)* (1)
	rep_cluster_2335 (1)	MOBP (1)	—	—	mobilizable	*qnrS1* (1)

Relaxase type refers to the relaxase enzyme initiating plasmid transfer. MPF type represents the mating pair formation system involved in conjugation. oriT type denotes the origin of transfer sequence on the plasmid. n represents the count of plasmids or resistance genes within the corresponding *Salmonella* serovars.

**Table 3:** HierCC clustering of *Salmonella* isolates and estimation of divergence times.

Seortype	HierCC Cluster	Number of Isolates	MRCA	R^2^
Enteritidis	HC20-87	250	1963.2 ± 3.82	0.68
Enteritidis	HC20-374	52	2005.7 ± 2.27	0.35
Enteritidis	HC20-7228	63	2000.0 ± 2.87	0.74
Enteritidis	HC20-10145	25	2004.6 ± 2.66	0.78
Enteritidis	HC50-87	249	1952.2 ± 5.99	0.64
Infantis	HC20-343	124	2008.6 ± 0.78	0.73
Infantis	HC50-36	161	1957.8 ± 6.32	0.20
London	HC50-37	77	1964.0 ± 4.66	0.33
Kentucky	HC50-528	104	1994.3 ± 3.13	0.57
Rissen	HC50-142	80	1983.2 ± 3.35	0.68
Indiana	HC50-1290	40	1985.2 ± 3.96	0.43
Agona	HC20-419	63	1967.8 ± 4.70	0.77
Agona	HC50-29	80	1942.0 ± 8.19	0.64
Typhimurium	HC50-2304	30	1993.7 ± 1.93	0.90
I 1,4,[5],12:i:-	HC20-2	137	1987.4 ± 7.63	0.54
Newport	HC50-3472	38	1980.9 ± 8.48	0.50
Thompson	HC20-11098	19	1978.2 ± 26.92	0.44
Thompson	HC50-119	46	1918.4 ± 20.16	0.51
I B:e,h:-	HC20-1406	66	1999.4 ± 2.97	0.15
Corvallis	HC50-70	157	1981.1 ± 4.11	0.55
Goldcoast	HC20-13193	28	1995.1 ± 4.69	0.36
Mbandaka	HC20-4	52	1957.4 ± 11.59	0.38
Muenster	HC50-47	40	1960.8 ± 32.91	0.44
Schwarzengrund	HC50-54	20	1936.2 ± 15.63	0.72
Stanley	HC50-8074	13	1985.1 ± 4.39	0.31
Weltevreden	HC50-314	74	1897.1 ± 32.83	0.63

MRCA: Most recent common ancestor of isolates within the cluster. R^2^: coefficient of determination from root-to-tip regression analysis.

## Data Availability

The original contributions presented in this study are included in the article and Supplementary Materials. Further inquiries can be directed to the corresponding authors.

## Supplementary Materials

The following supporting information can be downloaded at: https://www.mdpi.com/article/10.3390/microorganisms14010191/s1, Table S1: Accession numbers of sequenced *Salmonella* isolates; Table S2: Accession numbers of publicly available *Salmonella* isolates analyzed; Table S3: MPN of *Salmonella* isolates; Table S4: Antimicrobial resistance of *Salmonella* isolates; Table S5: Antimicrobial resistance genes of *Salmonella* isolates; Table S6: Correlations between antibiotic resistance phenotypes and antimicrobial resistance genes; Table S7: Plasmid replicon types of *Salmonella* isolates. Table S8: Completeness and contamination assessment of assembled *Salmonella* genomes.
